# Inadequate Awareness among Chronic Kidney Disease Patients Regarding Food and Drinks Containing Artificially Added Phosphate

**DOI:** 10.1371/journal.pone.0078660

**Published:** 2013-11-13

**Authors:** Yoshiko Shutto, Michiko Shimada, Maiko Kitajima, Hideaki Yamabe, Yoko Saitoh, Hisao Saitoh, Mohammed S. Razzaque

**Affiliations:** 1 Department of Health Promotion, Hirosaki University Graduate School of Health Sciences, Hirosaki, Japan; 2 Department of Cardiology, Respiratory Medicine and Nephrology, Hirosaki University Graduate School of Medicine, Hirosaki, Japan; 3 Oyokyo Kidney Research Institute Hirosaki Hospital, Hirosaki, Japan; 4 Department of Oral Medicine, Infection & Immunity, Harvard School of Dental Medicine, Boston, Massachusetts, United States of America; University of Sao Paulo Medical School, Brazil

## Abstract

Hyperphosphatemia is an important determinant of morbidity and mortality in patients with chronic kidney disease (CKD). Patients with CKD are advised to consume a low phosphate diet and are often prescribed phosphate-lowering drug therapy. However, commercially processed food and drinks often contain phosphate compounds, but the phosphate level is not usually provided in the ingredient list, which makes it difficult for CKD patients to choose a correct diet. We conducted a survey of the awareness of food/beverages containing artificially added phosphate among CKD patients undergoing hemodialysis. The subjects were 153 patients (77 males and 76 females; average age 56±11 years) who were randomly selected from the Dialysis Center of Hirosaki City, Japan. The subjects were provided with a list of questions. The survey results showed that 93% of the subjects were aware of the presence of high sugar content in soda, whereas only 25% were aware of the presence of phosphate (phosphoric acid) in such drinks. Despite 78% of the subjects being aware of the detrimental effects of consumption of a high phosphate diet, 43% drank at least 1 to 5 cans of soda per week and about 17% consumed “fast food” once each week. We also assessed the immediate effects of high-phosphate containing carbonated soda consumption by determining urinary calcium, phosphate, protein and sugar contents in overnight fasted healthy volunteers (n = 55; average age 20.7±0.3 years old, 20 males and 35 females). Significantly higher urinary calcium (adjusted using urinary creatinine) excretion was found 2 h after consuming 350 ml of carbonated soda compared to the fasting baseline level (0.15±0.01 vs. 0.09±0.01, p = 0.001). Our survey results suggest that CKD patients undergoing hemodialysis are not adequately aware of the hidden source of phosphate in their diet, and emphasize the need for educational initiatives to raise awareness of this issue among CKD patients.

## Introduction

Dysregulation of phosphate balance can affect the functionality of most organ systems [1], [2], [3], [4], [5], [6], [7], [8], [9]. Several studies have shown that hyperphosphatemia is the single most important determinant of mortality in CKD patients undergoing hemodialysis [10], [11], perhaps due to cardiovascular damage [12], [13], [14]. Excessive retention of phosphate can cause extensive cellular and tissue damage, and a higher incidence of vascular calcification related to hyperphosphatemia is found in patients with CKD [1], [9], [15]. Phosphate toxicity has been associated with cardiovascular calcification in various genetically modified mouse models [16], [17], [18], [19]. More importantly, reduction of serum phosphate levels in these models can markedly suppress vascular calcification, despite the presence of significantly higher serum calcium and 1,25- dihydroxyvitamin D levels [9], [16]. In patients with CKD, hyperphosphatemia and low serum vitamin D levels are independent risk factors for high mortality [20], [21], [22]. Therefore, reducing serum phosphate in patients with CKD is a therapeutic priority, and these patients are often asked to consume a low-phosphate diet, in addition to taking phosphate-lowering drugs. Hence, it is important for CKD patients to be aware of food items and beverages that are rich in artificially added phosphates.

Organic and inorganic forms of phosphate are present in meat, fish, eggs, milk/dairy products, and vegetables. These foods are required for maintaining normal nutritional balance and avoidance of these foods is not a suitable option. However, CKD patients may need to reduce intake of some of these food items as required to minimize phosphate intake. Of particular importance, processed food items and beverages are rich in phosphate. Therefore, total phosphate ingestion is likely to be increased significantly by consumption of processed food and soda.

Developing awareness of food items and drinks containing artificially added phosphate is becoming more important because of widespread use of phosphate as a preservative in processed food items, which complicates the ability of CKD patients to reduce phosphate intake. To assess the level of awareness of CKD patients regarding food and drinks containing artificially added phosphate, we conducted a survey of CKD patients undergoing hemodialysis in multiple dialysis centers in Hirosaki, Japan. We also assessed the immediate effects of consumption of carbonated soda with a high-phosphate content in overnight fasted healthy volunteers by determining urinary calcium, phosphate, protein and sugar contents before and 2 hours after consumption of 350 ml of soda.

## Methods

### Survey population

The subjects were 153 randomly selected CKD patients (77 males, 76 females) undergoing hemodialysis who are currently enrolled at the Dialysis Center in Hirosaki City, Japan. The average age of the subjects was 56±11 years old. All patients participated voluntarily on the assurance of anonymity, and none refused to answer the survey questions. A questionnaire consisting of seven questions was used to assess each patient's level of awareness about food and drinks containing artificially added phosphate (**Table S1**). The survey was conducted in June 2011. Informed consent was collected from each subject. All participation was on a volunteer basis and no financial compensation was offered. The results of the survey were analyzed using Microsoft Excel 2007 and IBM SPSS Statistics 19. A Mann-Whitney U test was used for comparison between groups.

### Effects of consumption of carbonated soda

The subjects in this study were 55 Hirosaki University students (average age 20.7±0.3 years old; 20 males, 35 females) who were randomly divided into a group that drank 350 ml of carbonated soda (n = 35; average age 21.2±0.4 years old; 13 males, 22 females) and a control group (n = 20; average age 19.9±0.3 years old; 7 males, 13 females) who drank 350 ml of water, both after an overnight fast. Urine samples were collected before and 2 hours after drinking the soda or water. Urinary protein contents and glucose were determined qualitatively in both samples of urine using test paper, and the amounts of urinary phosphate, calcium and creatinine were measured in both samples using an auto-analyzer. The urinary phosphate and calcium levels were normalized using the urinary creatinine concentration. Data were analyzed using a Wilcoxon signed-rank test and a Pearson correlation test, with a value of p<0.05 considered to be significant. The survey and the carbonated soda study were approved by the Committee for Medical Ethics of Hirosaki University, Japan.

## Results and Discussion

**Figure 1:**
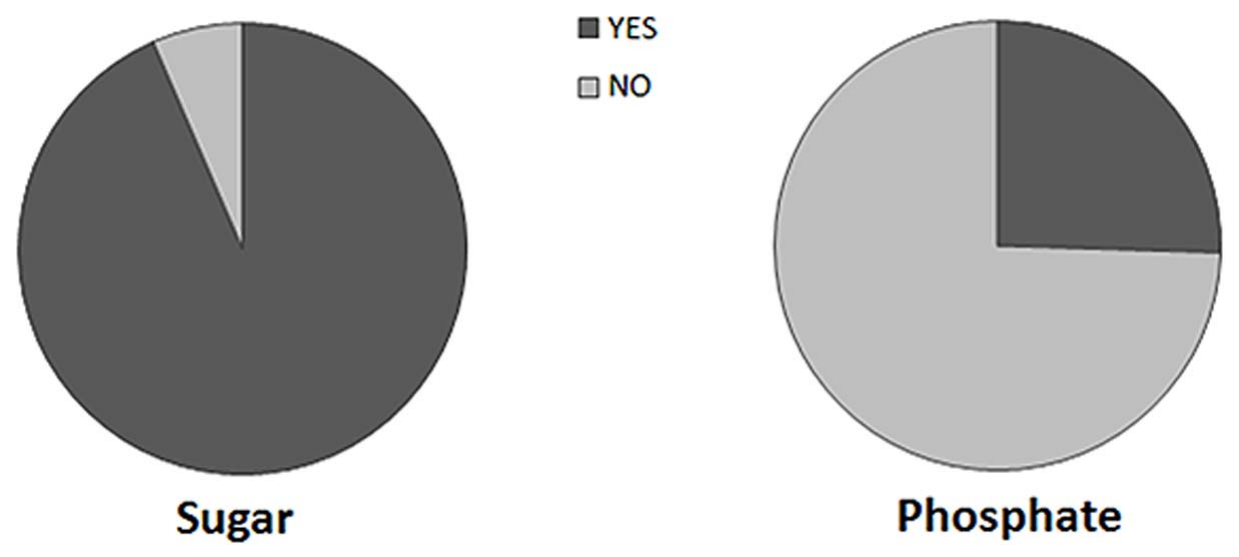
The survey participant CKD patients were asked whether they were aware of the sugar and phosphate content in commercially available soda drinks. Almost 93% of the participants were aware of the presence of sugar, while only 25% were aware of the presence of phosphate (phosphoric acid) in such drink, showing a noticeable awareness gap related to phosphate-contenting drinks among the patients.

To determine the level of awareness of food and drinks containing artificially added phosphate, we conducted a survey on CKD patients undergoing hemodialysis at the Dialysis Center in Hirosaki City, Japan. Since patients with CKD are required to maintain a low-phosphate diet, it is important that they are fully aware of food items (hamburgers and pizza) and beverages (soda) that contain artificially added phosphate. Based on this survey, 93% of the CKD patients were aware of the high sugar content in soda, but only 25% were aware of the presence of phosphate (phosphoric acid) in similar drinks. Similarly, 46% of the CKD patients were not aware that phosphate, in the form of a preservative, is routinely added to processed foods such as hamburgers and pizza (**Figure 1**). The survey results, categorized by gender, are shown in **Table S2**. It is clear from the survey that the CKD patients were often uninformed about the phosphate content in food and drinks, and therefore did not understand the detrimental effect of consuming such items.

**Figure 2:**
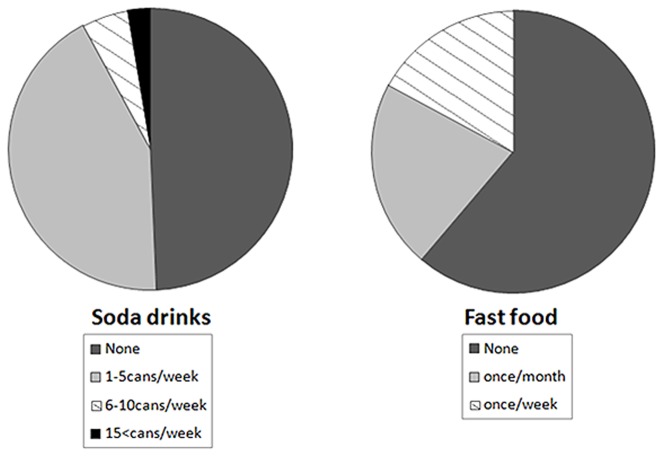
The participants were asked to describe their soda drink and fast food consumption habits. Almost half (51%) of the CKD patients drink soda, while 39% patients eat fast food.

More importantly, despite 78% of the surveyed patients being aware of the detrimental effects of consumption of a high phosphate diet, 43% drank at least 1 to 5 cans of soda per week, while another 5% consumed 6 to 10 cans of soda per week. About 17% of the surveyed patients ate fast food (hamburgers, pizza, etc.) once each week and another 22% consumed these items once each month (**Figure 2**). The survey results categorized by age groups are shown in **Table S3**.

**Figure 3:**
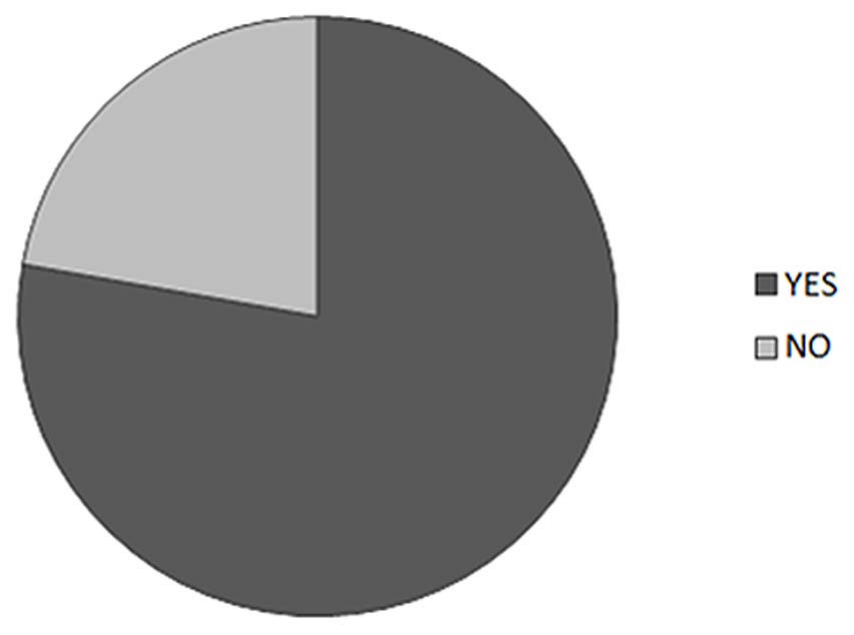
The survey participant CKD patients were asked whether they were aware of the possible harmful effects of unrestricted consumption of a high phosphate diet, and the majority (78%) of the participants was aware of detrimental effects related to high phosphate diet.

The majority (78%) of the CKD patients in the survey did appreciate the risk of consuming high phosphate-containing food and drinks (**Figure 3**). These responses differed significantly from those of medical and nursing students, since only 32% of the students were aware of the risks associated with uncontrolled phosphate consumption [23]. Despite 87% of the CKD patients recognizing the impact of phosphate on disease progression, only 25% were aware that most soda contains a phosphate compound (phosphoric acid).

**Figure 4:**
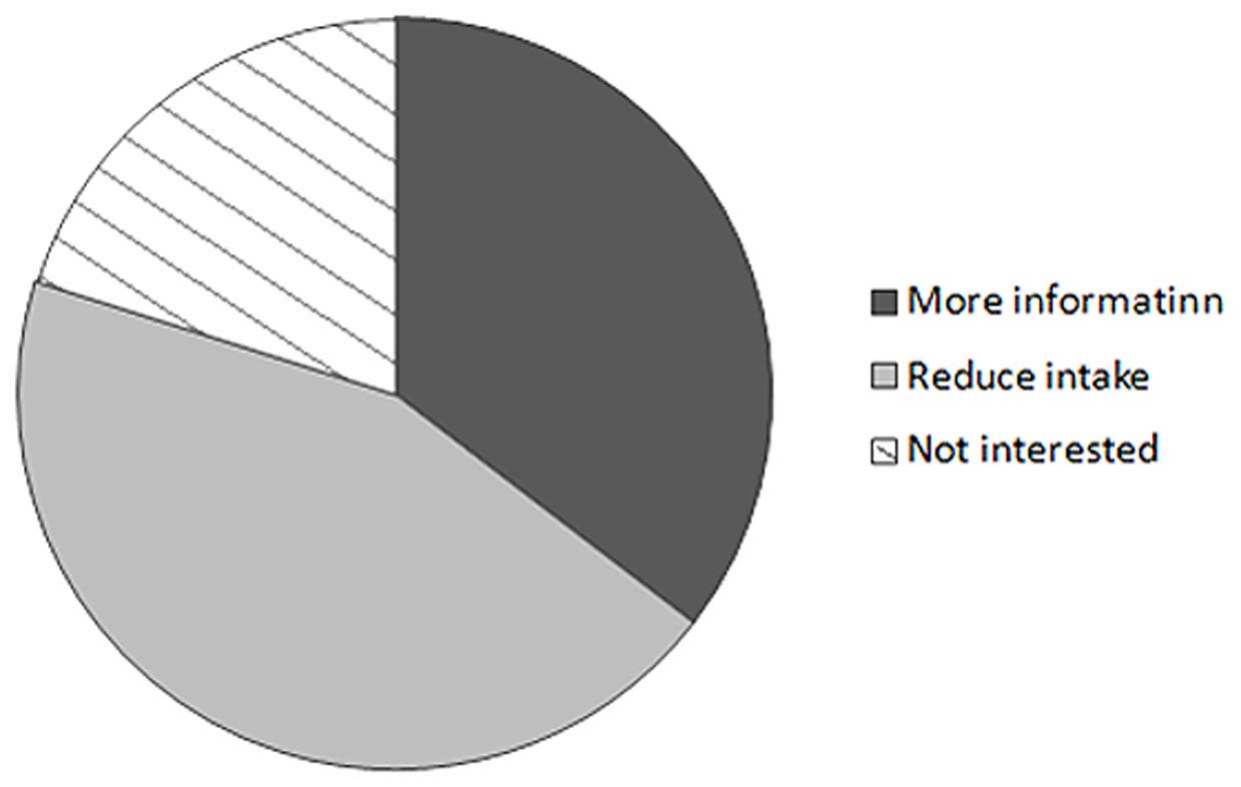
The survey participant CKD patients were asked whether they were willing to modify their diet to reduce phosphate intake. Around 35% of the participants wanted to have more information related to artificially containing-food and drinks, and another 45% were willing to reduce their phosphate intake by minimizing consumption of processed food and soda drinks.

The results of the survey clearly suggest that many CKD patients lack awareness of the presence of phosphate in food and drinks. However, after explaining this issue to the patients, 35% were eager to obtain information related to food containing phosphate and 45% were willing to consider reducing their phosphate intake by minimizing consumption of processed food and soda (**Figure 4**). These findings show the potential benefits of education of patients. Such educational guidance of CKD patients to make healthier food choices is likely to help in reducing complications related to abnormal mineral ion metabolism [24], [25], [26], [27], which is particularly important, given that the global prevalence of CKD may be as high as 500 million [28].

Notably, almost half of the surveyed Japanese patients did not consume any soda (**Figure 2**). One reasons for this result may be their strong preference for green tea, since almost 87% of Japanese adults (40 to 69 years old; n = 13,916) have been found to consume green tea every day [29]. Therefore, availability of an alternative healthy drink may reduce consumption of soda with high levels of sugar and phosphate.

The awareness of a majority (78%) of the subjects regarding effects related to a high-phosphate diet (**Figure 3**) indicates that healthcare providers, including doctors, nurses, and nutritionists, have been effective in informing patients of phosphate-related complications. However, an important finding in the survey was that about half (51%) of the CKD patients drank soda and 39% ate fast food (**Figure 2**), despite the high level of awareness of phosphate-related harmful effects. This may be explained by the fact that 75% of the surveyed patients did not realize that carbonated soda contains high levels of phosphate (**Figure 1**). Similarly, half of the surveyed patients were not aware of the high phosphate content in fast food and processed food items.

In summary, our results make it clear that there is an awareness gap among CKD patients regarding phosphate-containing foods and drinks. An issue that needs further evaluation, but is not within the scope of this survey, is the level to which healthcare providers are informing patients of the risk of a high-phosphate diet without providing sufficient details of the kind of foods and drinks that contain high phosphate. The results of this survey highlight a gap between the patients and healthcare providers, in terms of education of patients on foods with high phosphate levels. Of particular importance, the absence of listing of phosphate content in the nutritional ingredients makes it harder for patients to avoid food containing higher levels of phosphate.

A limitation of the study is that most of the surveyed patients drank soda and/or ate fast food due to lack of awareness of the phosphate content, and therefore we did not have enough patients to form a control group of non-consumers of these products. However, studies in overnight-fasted healthy volunteers show excretion of significantly increased amounts of urinary calcium at two hours after drinking soda, suggesting that the high phosphate content in the drink can influence other mineral metabolism. In the current study, the mean level of urinary calcium (adjusted using urinary creatinine) in 35 overnight fasted volunteers increased significantly from before to 2 hours after consuming 350 ml of carbonated soda (0.09±0.01 vs. 0.15±0.01, p = 0.001). In contrast, there was no such change in urinary calcium excretion from before to after drinking water in 20 control subjects. Urinary phosphate levels (adjusted using urinary creatinine) showed a slight, but not significant, increase from before to 2 hours after soda consumption (0.33±0.03 vs. 0.36±0.03, p = 0.252), and was unchanged in samples collected from the controls. A urinary analysis did not detect abnormal excretion of urinary protein and sugar in samples collected before and after drinking soda or water (data not shown).

The long-term effects of soda consumption on mineral ion metabolism were not examined in this study. This issue requires further study to show that increased serum phosphate levels in patients consuming fast food and soda are not harmless, and that the high-phosphate diet cannot be justified. In fact, it is becoming more evident from experimental and human studies that features of phosphate toxicity can appear after consumption of a high-phosphate diet, even when serum phosphate levels are within the normal range [30], [31].

The results of the survey highlight two important points. First, CKD patients are not sufficiently aware of food items and drinks that contain artificially added phosphates. Second, there is a need for an educational initiative to raise awareness of the risks posed by dietary items with hidden phosphate content.

## Supporting Information

Table S1 **List of Questioner.**
(DOC)

Table S2 **Survey results categorized by gender.**
(DOC)

Table S3 **Survey results categorized by different age groups.**
(DOC)
