# Supplementary material for: Inadequate Awareness among Chronic Kidney Disease Patients Regarding Food and Drinks Containing Artificially Added Phosphate
Source: PLoS One. 2013 Nov 13;8(11):e78660. doi: 10.1371/journal.pone.0078660 (PMC3827266; doi:10.1371/journal.pone.0078660)
Supplement: Table S2 — Survey results categorized by gender. (DOC) [file pone.0078660.s002.doc]

| n=153 | Male n=77 | Female n=76 | P value |
| --- | --- | --- | --- |
| Age | 56.3±9.4 | 56.4±11.6 |  |
| Q1 |  |  |  |
| YES | 72(93.5%) | 71(93.4%) | 0.98 |
| NO | 5(6.5%) | 5(6.6%) |
| Q2 |  |  |  |
| YES | 21(27.3%) | 18(23.7%) | 0.61 |
| NO | 56(72.7%) | 58(76.3%) |
| Q3 |  |  |  |
| YES | 40(51.9%) | 47(61.8%) | 0.22 |
| NO | 37(48.1%) | 29(38.2%) |
| Q4 |  |  |  |
| YES | 59(76,6%) | 60(78.9%) | 0.73 |
| NO | 18(23.4%) | 16(21.1%) |
| Q5 |  |  |  |
| 1 | 36(46.8%) | 39(52.0%) | 0.2 |
| 2 | 32(41.6%) | 33(44.0%) |
| 3 | 5(6.5%) | 3(4.0%) |
| 4 | 4(5.2%) | 0(0.0%) |
| Q6 |  |  |  |
| 1 | 46(60.5%) | 47(61.8%) | 0.14 |
| 2 | 13(17.1%) | 20(26.3%) |
| 3 | 17(22.4%) | 9(11.8%) |
| Q7 |  |  |  |
| 1 | 21(28.8%) | 30(42.3%) | <0.05 |
| 2 | 31(42.5%) | 33(46.5%) |
| 3 | 21(28.8%) | 8(11.3%) |
