# Supplementary material for: Inadequate Awareness among Chronic Kidney Disease Patients Regarding Food and Drinks Containing Artificially Added Phosphate
Source: PLoS One. 2013 Nov 13;8(11):e78660. doi: 10.1371/journal.pone.0078660 (PMC3827266; doi:10.1371/journal.pone.0078660)
Supplement: Table S3 — Survey results categorized by different age groups. (DOC) [file pone.0078660.s003.doc]

| n=153 | Under 60 n=75 | Over 60 n=78 | P value |
| --- | --- | --- | --- |
| Male | 38 | 39 |  |
| Female | 37 | 39 |  |
| Q1 |  |  |  |
| YES | 72(96.0%) | 71(91.0%) | 0.21 |
| NO | 3(4.0%) | 7(9.0%) |
| Q2 |  |  |  |
| YES | 23(30.7%) | 16(20.5%) | 0.15 |
| NO | 52(69.3%) | 62(79.5%) |
| Q3 |  |  |  |
| YES | 58(77.3%) | 29(37.2%) | <0.001 |
| NO | 17(22.7%) | 49(62.8%) |
| Q4 |  |  |  |
| YES | 66(88.0%) | 53(67.9%) | <0.01 |
| NO | 9(12.0%) | 25(32.1%) |
| Q5 |  |  |  |
| 1 | 36(48.6%) | 39(50%) | 0.29 |
| 2 | 29(39.2%) | 36(46.2%) |
| 3 | 6(8.1%) | 2(2.7%) |
| 4 | 3(4.1%) | 1(1.3%) |
| Q6 |  |  |  |
| 1 | 30(40.5%) | 63(80.8%) | <0.001 |
| 2 | 24(32.4%) | 9(11.5%) |
| 3 | 20(27.0%) | 6(7.7%) |
| Q7 |  |  |  |
| 1 | 16(23.2%) | 35(46.7%) | <0.001 |
| 2 | 45(65.2%) | 19(25.3%) |
| 3 | 8(11.6%) | 21(28.0%) |
